# Effect of a nurse-led integrated care intervention on quality of life and rehospitalisation in patients with severe exacerbation of COPD—a pilot study

**DOI:** 10.1177/14799731241291067

**Published:** 2024-10-15

**Authors:** Gabriela Schmid-Mohler, Christine Hübsch, Julia Braun, Claudia Steurer-Stey, Celine Aregger, Dominik J. Schaer, Christian Clarenbach

**Affiliations:** 1 Centre of Clinical Nursing Science, 27243 University Hospital Zurich, Zurich, Switzerland; 2 Division of Pulmonology, 27243 University Hospital Zurich, Zurich, Switzerland; 3 Epidemiology, Biostatistics and Prevention Institute, University of Zurich, Zurich, Switzerland; 4 MediX Group Practice Zurich, Zürich, Switzerland; 5 Physiotherapy Occupational Therapy, 27243 University Hospital Zurich, Zurich, Switzerland; 6 Division of Internal Medicine, 27243 University Hospital Zurich, Zurich, Switzerland; 7 Faculty of Medicine, 27217 University of Zurich, Zurich, Switzerland

**Keywords:** Patient readmission, self-management, nursing, COPD, quality of life, pilot studies

## Abstract

**Objective:**

To explore the feasibility and effect of a nurse-led integrated care intervention on health-related quality of life (QoL) and unplanned 90-day rehospitalisation in patients hospitalised due to acute exacerbation of COPD (AECOPD).

**Method:**

A monocentric non-randomized parallel cluster design was applied. The primary endpoint was the difference between Chronic Respiratory Questionnaire (CRQ) Mastery Scores at hospital discharge and 13 weeks post-discharge. Secondary endpoints were differences between other CRQ scores, numbers of rehospitalisations and self-reported exacerbations. The study would end either 13 weeks after the full sample size was achieved or when study time ran out.

**Results:**

The study was terminated before reaching the targeted sample size. Of 174 invitees, 69 (39.7%, 30 control, 39 intervention) consented to participate. Of those, 47 completed the study, 45 of whom had complete data sets for the primary endpoint. No differences in QoL scores, unplanned COPD-related rehospitalisations or unplanned all-cause rehospitalisations were detected. The mean number of self-reported moderate exacerbations was higher in the intervention group (*p* = 0.006).

**Conclusion:**

The pilot study had slow recruitment, high drop-out rates, and no significant effect on 3-month outcomes. Further research should focus on enhancing the current understanding of how to motivate and recruit patients in this setting.

**ClinicalTrials.gov ID:**

NCT 04011332.

## Introduction

COPD patients generally experience one to two pulmonary exacerbations annually, potentially requiring hospitalisation.^1^ Acute exacerbations of COPD (AECOPD) are intrusive and emotionally burdensome^2^: they often affect not only pulmonary function, but long-term COPD prognoses, including mortality risk.^1,3^ Additionally, they are expensive: 60%–70% of all COPD health costs result from exacerbation-linked hospitalisations.^3^ Improved outcomes, for example, lower numbers of exacerbations and hospitalisations, as well as higher health-related quality of life (QoL), often reflect changes in patients’ health behaviours, especially smoking cessation, increased physical activity and symptom management.^4^ Therefore, self-management support^5–8^ and integrated care components^9,10^ are key treatment elements.

While diagnostic and therapeutic procedures typically meet high standards, minimal in-hospital interventions are implemented to promote health-improving behaviours.^11–13^ Further, facilitators and barriers to interventions’ implementation are poorly understood.

Therefore, a 13-week nurse-led integrated care intervention was developed. Featuring integrated care components embedded in a Behaviour Change Wheel-based self-management component,^14^ this specifically addresses AECOPD inpatients. Recipients are followed up, mainly by phone, for 13 weeks by advanced practice nurses (APNs).

The intervention focuses strongly on improving key health behaviours and harmonizing treatment plans between patients’ family doctors and/or pulmonologists. Hospital healthcare professionals assessed it as acceptable, appropriate and feasible.^15^ However, from the patients’ perspective, neither the intervention’s nor the study’s feasibility was established. Before a multi-centre intervention study is possible, both will require confirmation. This study’s aim was to gain first insights regarding the intervention’s feasibility and effects, especially regarding QoL and rehospitalisation.

## Method

### Design and study population

**Figure 1.**
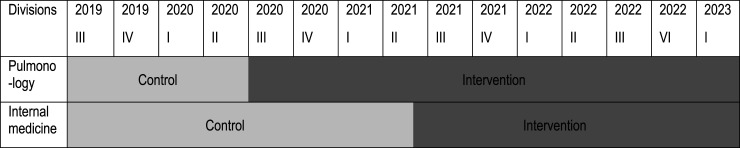
Study process on cluster level.

This pilot study used a monocentric non-randomized parallel cluster design with a baseline period. This design allows the implementation of scientific evidence into daily patient care, which is a fundamental aim of implementation science.^16^ The two parallel clusters were the two participating divisions (Pulmonology and Internal Medicine). Allocation to control and intervention groups was based on time. The intervention was introduced in University Hospital Zurich’s Divisions of Pulmonology (June 2020–March 2023) and Internal Medicine (May 2021–March 2023). Intervention participants’ data were compared to those of the control group (baseline period) (Pulmonology: July 2019–June 2020; Internal Medicine: July 2019–April 2021) (Figure 1).

The study had two possible endpoints: 1. 13 weeks after the optimal sample size (based on power calculations) was achieved (*n* = 64 per group, total *n* = 128); or 2. When study time ran out (13 weeks after 31 December, 2022, the last possible patient inclusion date). Study and intervention feasibility ratings both depended on the percentage of patients who completed them within the available period.

Inclusion criteria were age ≥18, a spirometry-confirmed COPD diagnosis, and hospitalisation on the Division of Pulmonology or Internal Medicine for AECOPD treatment using steroids and/or antibiotics. Exclusion criteria were cognitive impairment (scores >3 on the Delirium Observation Scale, >6 on the Alcohol Withdrawal Syndrome Scale, or <3 on the Mini-Cog dementia assessment), previous participation in the same study group, or inability to speak German, French, Italian, English, Spanish, Portuguese, Serbian, Tamil, Hindi, Turkish or Slovakian.

The Ethics Committee of the Canton of Zurich, Switzerland granted ethical approval (KEK ZH-Nr. 2019-00797).

### Study intervention

As is common in Switzerland, the control group received usual care as described by Markun and colleagues.^13^ This focuses strongly on acute inpatient needs, which are predominantly managed by physicians, ward nurses and physiotherapists. It normally offers no or very limited self-management support and involves a single follow-up visit at month three at the Division of Pulmonology outpatient clinic.

The intervention group received usual care (as described above) plus a 13-week nurse-led integrated care intervention focusing on seven key elements of COPD self-management: 1) physical activity; 2) acute symptom management with written action plans); 3) smoking cessation; 4) correct inhalation; 5) healthy diet to prevent under- or overweight; 6) advance care planning; and 7) management of illness-related emotional distress. Shortly after each participant’s admission, the responsible nurse and physiotherapist performed a comprehensive assessment. Depending on which elements were deficient, support interventions were delivered. Following an assessment of predefined criteria (e.g., cognitive skills, stable psychiatric condition, patient’s awareness of their COPD), an intervention nurse formulated an AECOPD action plan with oral steroids and/or antibiotics. The action plan was then sent to the patient’s physician, who reviewed it and prescribed any necessary medication adjustments. Post-discharge, the nurse continued the physiotherapist’s physical activity interventions.

On average, patients received ten counselling sessions over the 13-week study period—two in-hospital (face-to-face) and eight post-discharge as outpatients, mainly by telephone. The number of interventions was adapted based on each patient’s health state; so, patients reporting exacerbation symptoms received weekly phone calls. The nurses’ education and roles are explained in Supplement S1. The intervention is described in detail elsewhere.^14^

### Data collection

**Figure 2.**
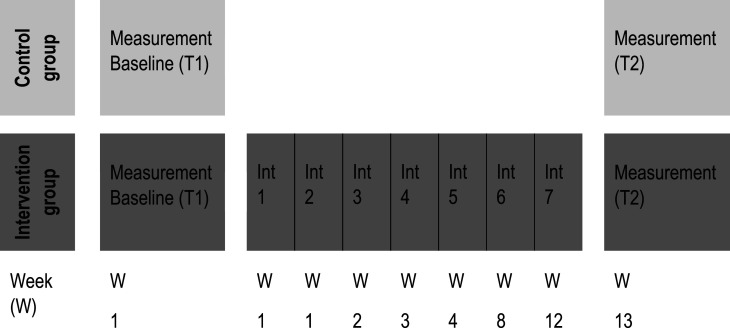
Study process for patients in the control and intervention phase.

Eligible patients were identified by reviewing electronic patient charts in the Divisions of Pulmonology and Internal Medicine. The study team contacted them and provided oral and written information about the study. For feasibility reasons, patients who declined study participation in the intervention phase were offered the intervention without study participation. For those who signed written informed consent, baseline measurement was performed by the study nurse. Data were gathered via an interview (for demographics and health behaviours) and several questionnaires (e.g., CRQ-SAS, CAT, mMRC). The second measurement, following the same procedure, took place in week 13 (Figure 2). Relevant clinical data were retrieved from the electronic patient charts.

### Variables

#### Health-related quality of life

Health-related quality of life was assessed at baseline and week 13 with the Chronic Respiratory Questionnaire–Self-Administered Standardised (CRQ–SAS).^17^ As the SAS version was developed for patients with chronic airflow limitations, it includes a set of standardised dyspnoea questions. Over a two-week recall period, its 20 items cover four domains: 1) dyspnoea, 2) fatigue, 3) emotional function and 4) mastery. The scoring scale ranges from one (extreme) to 7 (not at all). A sub-score can be calculated for each domain (sum of items/number of items). The CRQ-SAS is available in German, French, Italian, English, Spanish, Portuguese, Serbian, Tamil, Hindi, Turkish and Slovakian. In the evaluation of a rehabilitation programme, it showed high sensitivity to changes in health-related QoL.^18^

#### Rehospitalisations

The number of patients experiencing unplanned rehospitalisation during the study period (13 weeks) was assessed by patient report and validated using electronic patient file data. Unplanned COPD-related admissions were differentiated from all-cause rehospitalisations. Severe exacerbations were defined as emergency department visits or hospitalisations due to COPD symptoms, which were treated with steroids. As all emergency department visits led to rehospitalisations, all severe exacerbations led to unplanned COPD-related rehospitalisations.

#### Moderate exacerbations

Moderate exacerbations were assessed by patient report in the interview with the study nurse. Patients were asked the number of moderate exacerbations (treatable with steroids at home) during the study period (13 weeks).

#### Feasibility

Feasibility was assessed by participation rate in the study (overall, control and intervention group), study drop-out rates and number of patients who declined study participation, but consented to receive the intervention.

### Statistical analysis

#### Primary and secondary outcomes

The primary outcome was the difference between the CRQ Mastery Scores at discharge and 13 weeks post-discharge. Secondary outcomes were differences in the other CRQ scores (emotional functioning, dyspnoea and fatigue), number of unplanned all-cause and COPD-related rehospitalisations, and moderate exacerbations. For rehospitalisations, an intention to-treat-analysis was applied.

Sample characteristics were presented with descriptive statistical analysis. Group differences (control group (CG) versus intervention group (IG)) in the CRQ mastery score between baseline and month three were normally distributed and analysed with a two-sample *t* test. For continuous secondary outcomes, we also used a *t* test or a Wilcoxon test, plus a linear model adjusted for the baseline measurements. Group-level binary outcomes were compared using Fisher’s exact test. No imputation was performed for missing data. Statistical analyses were performed in R version 4.0.5.

For both the IG and the CG, a sample size of 64 was calculated to achieve 80% power to detect differences. Information on sample size calculations and the R software packages is provided in Supplement S2.

## Results

### Sample characteristics

**Figure 3.**
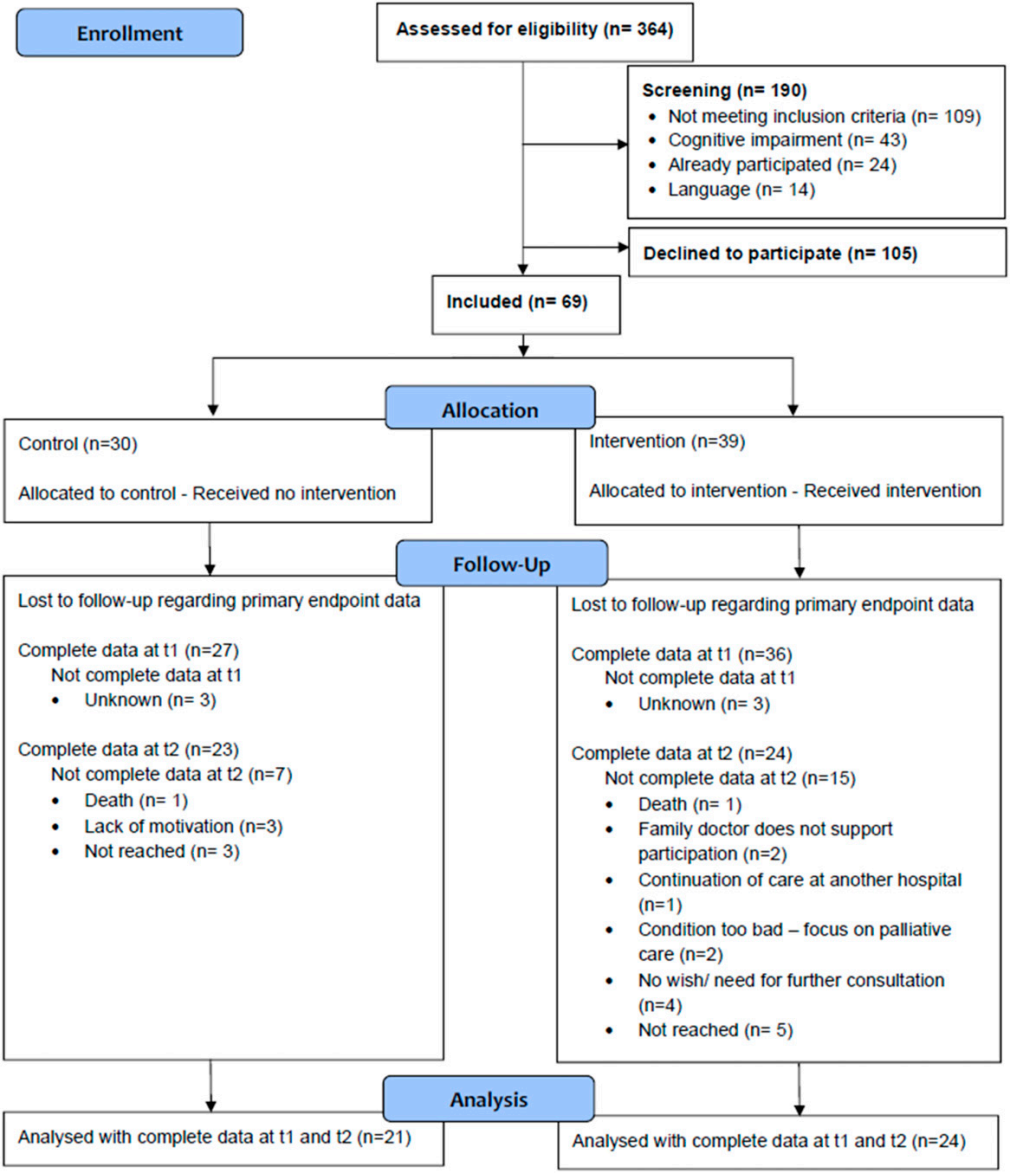
Flowchart.

The study was terminated 31 December 2022 without reaching the targeted sample size. Of 364 patients screened, 190 did not meet enrolment criteria and 105 declined. The remaining 69 (30 CG, 39 IG) were included. Of these, 47completed the study (drop-out rate = 31.9%), 45 of whom (21 CG, 24 IG) had complete data sets for the primary endpoint (Figure 3).

Sample characteristics at baseline measurement are listed in Table 1.

**Table 1.** Sample characteristics at baseline (*n* = 69).

Variable	Overall (*n* = 69)	Control (*n* = 30)	Intervention (*n* = 39)
Age, mean [SD]	69.2 [9.2]	69.6 [9.8]	68.9 [8.8]
Sex, *n* (%)			
Female	29 (42.0 %)	10 (33.3 %)	19 (48.7 %)
Male	40 (58.0 %)	20 (66.7 %)	20 (51.3 %)
Living alone, *n* (%)	27 (39.1 %)	16 (53.3 %)	11 (28.2 %)
Education, *n* (%)			
School not finished	2 (2.9 %)	1 (3.3 %)	1 (2.6 %)
School finished	4 (5.8 %)	2 (6.7 %)	2 (5.1 %)
Apprenticeship	49 (71.0 %)	20 (66.7 %)	29 (74.4 %)
University	11 (16.0 %)	6 (20.0 %)	5 (12.8 %)
missing	3 (4.3 %)	1 (3.3 %)	2 (5.1 %)
Nationality, *n* (%)			
Swiss	55 (79.7 %)	23 (76.7 %)	32 (82.1 %)
Other	13 (18.8 %)	6 (20.0 %)	7 (17.9 %)
Missing	1 (1.4 %)	1 (3.3 %)	0 (0.0 %)
Division, *n* (%)			
Pulmonology	60 (87.0 %)	23 (76.7 %)	37 (94.9 %)
Internal medicine	9 (13.0 %)	7 (23.3 %)	2 (5.1 %)
COPD GOLD stage, *n* (%)			
1	1 (1.4 %)	1 (3.3 %)	0 (0.0 %)
2	14 (20.3 %)	7 (23.3 %)	7 (18.0 %)
3	26 (37.7 %)	12 (40.0 %)	14 (35.9 %)
4	24 (34.8 %)	8 (26.7 %)	16 (41.0 %)
missing	4 (5.8 %)	2 (6.7 %)	2 (5.1 %)
FEV1 predicted %, mean [SD]	37.8 [17.0] (*n* = 61)	42.6 [18.4] (*n* = 25)	34.4 [15.3] (*n* = 36)
Long-term oxygen use, *n* (%)	49 (71.0%)	20 (66.7 %)	29 (74.4 %)
Had a written AECOPD action plan before hospitalisation	12 (17.4%)	7 (23.3%)	5 (12.8%)
Elements of action plan			
Informing the treating physician	9 (13.0%)	5 (16.7%)	4 (10.3%)
Increasing inhalation regimen	7 (10.1 %)	6 (20.0%)	1 (2.65)
Taking steroids/antibiotics	7 (10.1 %)	5 (16.7%)	2 (5.1%)
Number of self-reported moderate^a^ exacerbations in the last 12 months, *n* (%)			
0	38 (55.1 %)	13 (43.3 %)	25 (64.1 %)
1	15 (21.7 %)	7 (23.3%)	8 (20.5 %)
2	7 (10.1 %)	3 (10.0 %)	4 (10.3 %)
3	4 (5.8 %)	4 (13.3 %)	0 (0.0 %)
4	1 (1.4 %)	0 (0.0 %)	1 (2.6 %)
17	1 (1.4 %)	1 (3.3 %)	0 (0.0 %)
missing	3 (4.3 %)	2 (6.7 %)	1 (2.6 %)
Number of self-reported severe^b^ exacerbations in the last 12 months, *n* (%)			
0	1 (1.4%)	0 (0.0 %)	1 (2.6 %)
1	41 (59.4 %)	17 (56.7 %)	24 (61.5 %)
2	20 (29.0 %)	7 (23.3 %)	13 (33.3 %)
3	4 (5.8 %)	3 (10.0 %)	1 (2.6 %)
5	1 (1.4 %)	1 (3.3 %)	0 (0.0 %)
7	1 (1.4 %)	1 (3.3 %)	0 (0.0 %)
missing	1 (1.4 %)	1 (3.3 %)	0 (0.0 %)
COMCOLD^c^, mean [SD]	3.5 [3.7] (*n* = 54)	3.7 [4.2] (*n* = 21)	3.4 [3.4] (*n* = 33)
Number of comorbidities^d^, mean [SD]	3.0 [2.1] (*n* = 65)	3.3 [2.7] (*n* = 28)	2.9 [1.5] (*n* = 37)
BMI, mean [SD]	24.1 [5.6] (*n* = 61)	24.7 [3.7] (*n* = 25)	23.7 [6.6] (*n* = 36)
Smoking status, *n* (%)			
Still smoking	20 (29.0 %)	7 (23.3 %)	13 (33.3 %)
Never smoked	2 (2.9 %)	2 (6.7 %)	0 (0.0 %)
Stopped smoking	47 (68.1 %)	21 (70 %)	26 (66.7 %)
Pack years, mean [SD]	58.9 [37.4]	62.3 [44.5]	56.2 [31.2]
mMRC dyspnea scale^e^, mean [SD]	2.5 [1.1] (*n* = 53)	2.2 [1.1] (*n* = 19)	2.6 [1.0] (*n* = 34)
CAT score^f^, mean [SD]	21.5 [6.5] (*n* = 59)	20.8 [6.8] (*n* = 24)	22.1 [6.4] (*n* = 35)
HADS score^g^, mean [SD]			
Anxiety score	5.1 [3.8]	4.9 [4.0]	5.3 [3.7]
Depression score	6.6 [4.0] (*n* = 54)	5.7 [4.8] (*n* = 21)	7.1 [3.3] (*n* = 33)

Legend:

^a^Moderate: using steroids;

^b^Severe: hospitalization or emergency department visit and steroids;

^c^COMCOLD: Score ranges from 0 (no impact of comorbidity on health status) to 19 (very large impact of comorbidity on health status und includes depression, anxiety, peripheral arterial occlusive disease, cerebrovascular diseases, symptomatic heart disease22;

^d^Sum of total 23 comorbidities: 1. Diabetes, 2. Dyslipidaemia, 3. Arterial hypertension, 4. Ischemic heart disease/infarction, 5. Coronary arterial disease, 6. Systolic or diastolic dysfunction of the left ventricle, 7. Pulmonary hypertension, 8. Cor pulmonale, 9. Peripheral vascular disease, 10. Cerebrovascular disease, 11. Stroke, 12. Arrhythmia, 13. Obesity, 14. Cancer, 15. Sleep apnoea (obstructive), 16. Musculoskeletal disease, 17. Depression, 18. Anxiety disorder, 19. Cognitive disorder (dementia), 20. Anaemia, 21. Renal insufficiency, 22. Gastroesophageal reflux, 23. Thyroid gland disease;

^e^mMRC: The Modified Medical Research Council Dyspnoea Scale assesses dyspnoea ranging from 0 (dyspnoea only with very heavy exertion) to 4 (dyspnoea when getting dressed);

^f^CAT: The COPD Assessment Test assesses the impact of COPD on eight domains (cough, sputum, tightness, dyspnoea during activity, restriction in daily activities, sorrows to leave the house, sleep, energy) on a 5-point-Likert-Scale. The total score ranges from 0 (no impact of COPD) to 40 (heavy impact of COPD);

^g^HADS: The Hospital-Anxiety-and-Depression-Scale assesses Anxiety and Depression with 7 items each on a 4-point score (0-3). The sub-scores range from 0 (no symptoms) to 21 (heavy symptoms).

### Health-related quality of life

The mean mastery score improvement was 0.8 (SD 1.0) in the intervention and 0.7 (SD 1.5) in the control group (*p* = 0.76). No significant effect was detected in the other QoL scores (Table 2).

**Table 2.** CRQ scores at baseline and week 13 and differences on patient level.

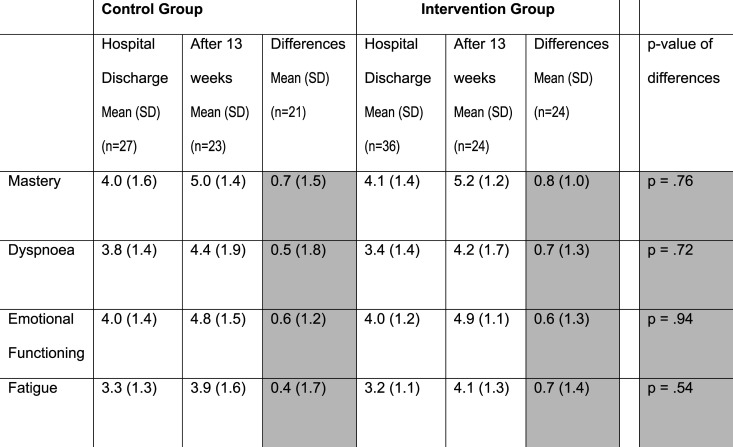

#### Unplanned rehospitalisations

*The* overall COPD-related unplanned rehospitalisation rate was 29.0% (IG: 33.3%; CG: 23.3%) (Table 3). 13 IG and 7 CG patients were rehospitalised for severe exacerbations (Fisher’s exact test *p* = 0.43). This difference was non-significant.

**Table 3.** Unplanned COPD-related rehospitalisations within 3 months after the index hospitalization (note: index hospitalization was also a COPD-related unplanned hospitalisation).

	Control *N* = 30	Intervention *N* = 39
Number of 1st COPD-related rehospitalisations N (%)	7 (23.3%)	13 (33.3%)
Number of 2nd COPD-related rehospitalisations N (%)	5 (16.7%)	4 (10.3%)
Number of 3rd COPD-related rehospitalisations N (%)	2 (6.7%)	1 (2.6%)
Total COPD-related rehospitalisations per group	14	18
Number of COPD-related rehospitalisations per patient	0.47	0.46

Also, while the median number of rehospitalisations per patient was three in the control group and 2 in the intervention group, a Mann-Whitney test *p*-value of 0.085 indicates that this difference is non-significant.

#### All-cause unplanned rehospitalisations

The all-cause unplanned rehospitalisation rate was 31.9% (*n* = 22) across all participants: 35.9% (*n* = 14) in the IG; 26.7% (*n* = 8) in the CG. Again, Fisher’s exact test (*p* = 0.45) indicates a non-significant result.

#### Moderate exacerbations

Twenty-two IG (43.3%, *n* = 30) and 2 CG patients (9.1%, *n* = 22) reported moderate exacerbations (Table 4). A Wilcoxon test of inter-group differences in self-reported moderate exacerbations yielded a *p*-value of 0.006, strongly suggesting an inter-group difference. IG patients self-reported a mean of 0.63 moderate exacerbations each, CG patients 0.09.

**Table 4.** Number of patient-reported moderate exacerbations between discharge and week 13 and written AECOPD action plans at month 3 (*n* = 69).

Variable	Overall (*n* = 69)	Control (*n* = 30)	Intervention (*n* = 39)
Number of self-reported moderate^a^ exacerbations in the last 3 months, *n* (%)			
0	37 (53.6 %)	20 (66.7 %)	17 (43.6 %)
1	10 (14.5 %)	2 (6.7 %)	8 (20.5 %)
2	4 (5.8 %)	0 (0.0 %)	4 (10.3 %)
3	1 (1.4 %)	0 (0.0 %)	1 (2.6 %)
missing	17 (24.6 %)	8 (26.7 %)	9 (23.1 %)
Received a written AECOPD action plan during intervention period	46 (66.7 %)	7 (23.3 %)	39 (100 %)
Elements of action plan			
Informing the treating physician	41 (59.4 %)	6 (20.0 %)	35 (89.7 %)
Increasing inhalation regimen	38 (55.1 %)	6 (20.0%)	32 (82.1 %)
Taking steroids/antibiotics	32 (46.4 %)	5 (16.7%)	27 (69.2 %)

Legend:

^a^Moderate: using steroids.

At month 3, 100% of IG and 23.3% of CG patients had written AECOPD action plans (Table 4).

#### Feasibility

The overall study participation rate was 39.7% (69/174) (CG: 44.8% (30/67); IG: 36.4% (39/107)). Reasons for non-participation were discharge before contact with study nurse (2 CG; 5 IG), living abroad (0 CG; 2 IG), feeling of being overburdened (18 CG; 3 IG), not interested (14 CG; 58 IG) and unreported (3 CG; 0 IG).

Of the 68 patients who declined study participation in the IG, 43 accepted an offer of the full or an adapted intervention without study participation. Consequently, the overall intervention participation rate was 76.6% (82/107), with 39 study and 43 non-study participants.

Of the 43 non-study participants, 21 (48.8%) took part in 1-3 interventions, which were mainly delivered pre-discharge. Seven (16.3%) received 4–7 interventions, eight (18.6%) received 8–12 interventions, and seven (16.3%) received 13–16.

Of the 39 study participants, nine (23.1%) received 4–7 interventions, twenty (51.3%) received the originally planned 8-12, and ten (25.6%) received 13–19.

Of the 82 intervention recipients, 61 (39 study participants, 22 non-study participants) accepted post-discharge follow-up interventions.

## Discussion

This pilot study did not show any significant relationship between the evaluated intervention and either rehospitalisation rates or QoL scores. Self-reported moderate exacerbations were more common in the IG. This pilot study encountered slow study recruitment and moderate study participation, which decreased during the intervention.

This study’s participation rate, that is, the participant/non-participant ratio of all eligible patients, was 39.7%. Normally, studies focusing on patients hospitalised due to COPD exacerbation report participation rates of 54.9%^19^–79%.^20^ The main reasons given for declining study participation were feelings of being overburdened and lack of interest. The latter explanation was more pronounced in the intervention group active between June 2020 and March 2023. As one explanation for this reduced interest, the simultaneous running of another study in the same population may have had a competing effect on study participation.

The drop-out rate, that is, the proportion of participants who left the study before all measurements were taken, was 34.8%. This may initially appear high, but is comparable to rates in other studies focusing on severe AECOPD. One reported a drop-out rate of 28.8% at month 3,^21^ another a rate of 41.1% at month 12.^19^ Only one of our reviewed studies (discussed below) reported a very low (7.1%) drop-out rate.^20^ In our study, patients’ lack of motivation, lack of desire for further consultation and lack of reachability by phone (possibly also reflecting motivational deficits) were the main reasons for drop-out. Patients’ motivation to participate may decrease when they experience either declining health conditions or worrying thoughts regarding health issues, either of which can shift priorities.^22^ We hope to address this dynamic in further program development by focusing on emotionally burdensome topics that are highly relevant from the patients’ perspective.

Considering that drop-out rates and reasons for drop-out were similar between IG and CG participants, we conclude that the intervention itself was not the reason for withdrawing. In fact, a substantial number of study decliners expressed interest in the intervention and three-quarters of eligible patients wished to receive all or part of the intervention, indicating a surprisingly high penetration rate.^23^ Half of the non-study-participants declined any post-discharge self-management interventions, yet accepted pre-discharge in-hospital counselling. Such selective acceptance highlights how lowering the threshold for participation can increase an intervention’s reach.

A recently presented pilot study using a single post-discharge counselling session yielded promising preliminary results concerning QoL and hospitalisations.^19^ It involves tracking key aspects of each patient’s life and thoughts, setting goals and documenting progress in an illustrated booklet. While it is unclear which, if any, of that intervention’s elements are effective, the Zurich Resource Model emphasizes that personal goal-setting and visualisation correlate with improved self-efficacy in asthma patients.^24^ Therefore, they will likely be part of our intervention’s further development.

The non-significance of our study’s QoL score changes may reflect our relatively short study period (3 months) and delivery of the intervention predominantly via phone rather than face-to-face. Two previous fully-powered RCTs of 3-month nurse-led interventions, both using the Saint George Respiratory Questionnaire to measure QoL in patients hospitalised due to AECOPD, showed conflicting results. While Aboumatar, Naqibuddin 21 found no significant changes in QoL, Wang, Zhao^20^ reported a positive effect. The disagreement may arise from dosage differences: While Aboumatar, Naqibuddin 21 COPD nurses delivered six sessions—three face-to-face, three by phone—Wang, Zhao^20^ APNs delivered 15: twelve weekly in-clinic sessions, plus three home visits. The Wang team’s more frequent direct contact may have fostered trust, a key element for patient support and motivation.^25^ Another possible source of disparity is adherence differences: Wang et al.’s group had exceptional participation rates and minimal (7.1%) drop-outs, suggesting outstandingly high adherence and motivation.

COPD-related and all-cause readmission rates were respectively 33.3% and 35.9% in our IG and respectively 23.3% and 26.7% in our CG. Our IG’s COPD-related readmission rates were lower than or comparable to those reported by other reliable, prospectively collected or registry-based data, which reported rates of 35.5%,^26^ 61%,^27^ and 56%.^28^ Regional practices also affect hospital admission rates. for example, some countries prefer homecare-based approaches to AECOPD; in Switzerland, emergency department visits for exacerbations commonly lead to hospitalisation. Such differences must be considered when comparing rehospitalisation rates.

However, studies of self-management interventions’ effects on AECOPD-related rehospitalisation rates commonly yield controversial results. For example, two retrospective studies reported strongly-decreased rehospitalisation rates: Truumees, Kendra^29^ reported a decrease from 43.8 % to 13.1% (*p*=<0.001) after delivering three respiratory therapist home visits over a 4-week period; and Kendra, Mansukhani^30^ reported a reduction from 32.2 % to 10.1 % (*p* > 0.001) in all-cause 90-day readmission after implementation of a post-discharge care-organization-focussed in-patient care bundle. The latter study’s retrospective design meant its rehospitalisation rates were underreported; however, the reported reduction apparently occurred following the intervention. The aforementioned RCTs provided wider views of intervention impacts on rehospitalisation rates: While Wang et al.^19^ reported decreased hospitalisation rates in their intervention group, Aboumatar, Naqibuddin ^21^ reported increases; and Atwood, Bhutani^26^ reported an all-cause 90-day readmission rate of 35.4% in patients already receiving a transition bundle. Following the addition of care coordinator consultations using a randomized design, this increased (non-significantly) to 35.5%.

As rehospitalisations result from diverse factors, reducing them requires complex interventions. While it is unknown which components are most effective, patients’ attitudes and social networks clearly play major roles.^31^ These must be addressed in further intervention development. Ospina et al.’s systematic review^32^ presented evidence that giving patients discharge care bundles including core-evidence-based interventions correlated with fewer hospital readmissions. However, sporadic delivery of such bundles^26^ left their benefits poorly-understood. Such failures underscore the complexity of implementing multi-professional interventions: before the implementation, each element must be tailored closely to the context; afterwards, the intervention’s uptake requires continuous supervision.^33^

Of our 30 intervention patients, thirteen (43.3%) reported at least one moderate exacerbation necessitating oral steroids during the intervention period, compared to 2 of 22 (9.1%) in the CG (*p* = 0.006 for both). This imbalance has several possible explanations: Firstly, patients in the intervention group received training to recognize and treat moderate exacerbation symptoms. For most, this included oral steroids. Secondly, the IG’s action plan implementation rate was 100% at month 3. IG patients were trained to keep a symptom journal, which was discussed in their weekly or bi-weekly phone calls. Therefore, we conclude that the CG’s low rate of treatment for moderate exacerbations reflected their inability to recognize exacerbation triggers and early warning symptoms.^34^

This study’s limitations include insufficient sample size, leading to a risk of a β type II error. Allocation to IG or CG was non-random. Also, the different time periods of data collection of the IG and the CG may bias the results; and the COVID pandemic influenced the number and characteristics of hospitalised patients. One further limitation was the relatively short study period. Behavioural change takes time, and its effects take time to produce detectable effects; therefore, measurable intervention effects regarding endpoints such as rehospitalisation and quality of life probably require a longer study period to develop.

This study provided first insights into the feasibility and effects of a nurse-led disease management intervention. The number of patients was not reached within the given time-frame, indicating the need for a multicentre approach in future. However, before attempting broader implementation and testing, further adaptation will be necessary, both to enhance the intervention’s reach in patients with COPD and to isolate its most effective elements. To enhance the reach of interventions targeting patients with COPD, further research is necessary to enhance our understanding of what patients perceive as valuable, welcoming and acceptable in interventions for persons experiencing severe AECOPD. In addition, an in-depth understanding is needed of how the intervention or its individual components affect outcomes and which are most effective. To achieve these aims, a combination of quantitative and qualitative research is recommended.^35^

## Supplemental Material

Supplemental Material - Effect of a nurse-led integrated care intervention on quality of life and rehospitalisation in patients with severe exacerbation of COPD—a pilot study Supplemental Material for Effect of a nurse-led integrated care intervention on quality of life and rehospitalisation in patients with severe exacerbation of COPD—a pilot study by Gabriela Schmid-Mohler, Christine Hübsch, Julia Braun, Claudia Steurer-Stey, Celine Aregger, Dominik J. Schaer and Christian Clarenbach in Chronic Respiratory Disease
