# Supplementary material for: Effect of a nurse-led integrated care intervention on quality of life and rehospitalisation in patients with severe exacerbation of COPD—a pilot study
Source: Chron Respir Dis. 2024 Oct 15;21:14799731241291067. doi: 10.1177/14799731241291067 (PMC11481074; doi:10.1177/14799731241291067)
Supplement: Supplemental Material - Effect of a nurse-led integrated care intervention on quality of life and rehospitalisation in patients with severe exacerbation of COPD—a pilot study [file sj-pdf-1-crd-10.1177_14799731241291067.pdf]

Supplement

Nurses' education and role

This study tested a thirteen-week nurse-led integrated care intervention. The intervention group involved a team of advanced practice nurses (APNs), as well as several physiotherapists and physicians. A PhD-prepared APN led the entire intervention nursing team. Each member of the intervention nursing team held a diploma; half also held masters' degrees in nursing science. All were clinically experienced in the care of patients with respiratory diseases and had special training in self-management support. This involved a basic training of at least two days, plus trimonthly one-to-one supervision in motivational interviewing.

The APN team managed patient care autonomously within the scope of practice agreed upon by the multi-professional team (including pulmonologists, nurses and physiotherapists). Throughout the thirteen-week study period, the APNs also coordinated the diverse involved health professionals (specialists, general practitioners, physiotherapists, nutritionists, etc.) both within the hospital and across transitions (e.g., hospital-rehabilitation, rehabilitation-home, hospital-home).

Sample Size Calculation

The sample size for the primary endpoint—CRQ mastery—was calculation based on the suggested *minimal clinically important difference* (MCID) of 0.5 points per sub-score (18) and the standard deviation (SD) of the difference between baseline and three-month scores determined by a meta-analysis of pulmonary rehabilitation studies (19). We calculated the SD from the various domains' standard errors (SEs), which ranged from 0.641 (for the dyspnoea and mastery domains (n=34)) (20) to 3.010 (for emotion domain (n=49)) (21). For this study, we assumed an SD of 1.0 for the difference between baseline and three-month scores for all four scores. We also calculated that a sample size of 64 for both IG and CG would achieve 80% power to detect 0.5-point differences between each of the four sub-scores and between the null hypothesis (that both group means would be 0.0) and the alternative hypothesis, i.e., that the mean of the intervention group would be 0.5, with an estimated SD of 1.0 across differences, using a significance level (alpha)—yielded by a two-sided two-sample t-test—of 0.05.

Statistical software packages used

Basic R software (stats, graphics, grDevices, utils, datasets, methods, base) and further packages (biostatUZH, survival, dplyr, stringr, reporttools, xtable, ggplot2, knitr) were used.
